# Factors Associated with *In Vitro* Fertilization Live Birth Outcome: A Comparison of Different Classification Methods

**DOI:** 10.22074/IJFS.2020.134582

**Published:** 2021-03-11

**Authors:** Payam Amini, Fariba Ramezanali, Mahta Parchehbaf-Kashani, Saman Maroufizadeh, Reza Omani-Samani, Azadeh Ghaheri

**Affiliations:** 1 Department of Biostatistics and Epidemiology, School of Public Health, Ahvaz Jundishapur University of Medical Sciences, Ahvaz, Iran; 2 Department of Endocrinology and Female Infertility, Reproductive Biomedicine Research Centre, Royan Institute for Repro- ductive Biomedicine, ACECR, Tehran, Iran; 3 Department of MBA, Payame Noor Tehran University, Tehran, Iran; 4 School of Nursing and Midwifery, Guilan University of Medical Sciences, Rasht, Iran; 5 Department of Medical Ethics and Law, Reproductive Biomedicine Research Centre, Royan Institute for Reproductive Bio- medicine, ACECR, Tehran, Iran; 6 Reproductive Epidemiology Research Centre, Royan Institute for Reproductive Biomedicine, ACECR, Tehran, Iran

**Keywords:** Assisted Reproductive Technology, Classification, Infertility, *In Vitro* Fertilization, Live Birth

## Abstract

**Background:**

*In vitro* fertilization (IVF) is a useful assisted reproductive technology to achieve pregnancy in infertile couples. However, it is very important to optimize the success rate after IVF by controlling for its influencing factors. This study aims to classify successful deliveries after IVF according to couples’ characteristics and available data on oocytes, sperm, and embryos using several classification methods.

**Materials and Methods:**

This historical cohort study was conducted in a referral infertility centre located in Tehran, Iran. The patients’ demographic and clinical variables for 6071 cycles during March 21, 2011 to March 20, 2014 were collected. We used six different machine learning approaches including support vector machine (SVM), extreme gradient boosting (XGBoost), logistic regression (LR), random forest (RF), naïve Bayes (NB), and linear discriminant analysis (LDA) to predict successful delivery. The results of the performed methods were compared using accuracy tools.

**Results:**

The rate of successful delivery was 81.2% among 4930 cycles. The total accuracy of the results exposed RF had the best performance among the six approaches (ACC=0.81). Regarding the importance of variables, total number of embryos, number of injected oocytes, cause of infertility, female age, and polycystic ovary syndrome (PCOS) were the most important factors predicting successful delivery.

**Conclusion:**

A successful delivery following IVF in infertile individuals is considerably affected by the number of embryos, number of injected oocytes, cause of infertility, female age, and PCOS.

## Introduction

*In vitro* fertilization (IVF) is considered a popular technique used in assisted reproductive technology (ART) to promote the achievement of childbirth in the population of infertile individuals. Numerous aspects of IVF treatments have changed over time. Substantial research has been conducted to improve IVF results by taking into consideration its influencing factors; however, there is still a lack of knowledge about the predictors of IVF outcomes while the overall pregnancy rates have only reached approximately 30% (1, 2).

Many factors have been known to affect IVF outcomes including age, sperm quality, fertilization rate, embryo quality, frequency of transferred embryos, and endometrial thickness (3, 4). Determining influencing factors, could potentially influence the likelihood for a successful IVF treatment; this would enable clinicians and physicians to make better decisions in order to apply IVF based on patients’ characteristics (5). Patients who failed treatments might experience adverse psychological problems such as depression and anxiety (6). Therefore, it is essential to assess factors associated with the outcome after IVF and determine the influencing factors. In order to reduce psychological and other negative outcomes after IVF, patients could evaluate the likelihood of successful IVF based on their characteristics.

Thus, machine learning approaches have been designed to assess the relationship of an outcome and its effective variables; The use of a hybrid intelligence method for knowledge exploring of a clinical database on IVF (7), an ordered mechanism in comparison with naïve Bayes (NB) classifier to estimate the odds of success after IVF (8), random forest (RF) and adaptive boosting in classifying the state of ART (9), and logistic regression (LR) to predict implantation after blastocyst transfer (10) are some examples of application of this approach on IVF data.

Here, we used a clinical database that included each couple’s characteristics and available data on oocytes, sperm and embryos, as well as the cycle outcomes to classify the IVF outcome (successful/unsuccessful delivery) by NB, RF, support vector machine (SVM), extreme gradient boosting (XGBoost), linear discriminant analysis (LDA), and LR.

## Materials and Methods

### Participants and study design

We conducted this historical cohort study in a referral infertility centre located in Tehran, Iran. Data from 6071 cycles performed during March 21, 2011 to March 20, 2014 were analysed. We included only those women for whom clinical pregnancy was confirmed observing an intrauterine gestational sac. The collected demographic and clinical variables comprised women’s ages, source of infertility (female factor, male factor, combined male-female factor infertility, unexplained), infertility type (primary, secondary), body mass index (BMI), infertility duration (years), number of previous abortions, polycystic ovary syndrome (PCOS), number of previous IVF attempts, total number of retrieved oocyte, number of injected oocytes, number of embryos, number of transferred embryos, spermogram, fertilization rate after intracytoplasmic sperm injection (ICSI), number of two-pronuclear embryos to number of metaphase II (MII) oocytes (2PN/MII ratio), and data on embryo quality (number of compact, blastocysts, grade A, grade AB, early blastocysts, A compact, and AB compact), as well as the day of the embryo transfer (ET). 

### Statistical analysis

The descriptive characteristics of the data are shown using mean (standard error) and frequency (percentage) for continuous and categorical variables, respectively. We used the independent samples t test after checking the normality of data distribution to compare the mean of the variables across the categories of the response. The chi-squaretest was used to assess the independence of categorical variables with the outcome.

A principle component approach was utilized to reduce the dimension of multiple independent variables into smaller components. To do so, the variables that included the numbers of compact, blastocyst, grade A, grade AB, early blastocyst, A compact and AB compact, and the day of the ET were entered in the principle component analysis. The best number of components is decided according to the highest determined variance of the variables so that the majority of variability in the independent variables is available in the result antcomponents.

For the classification approaches, we randomly divided the data into two sets of train (70%) and test (30%). The train set was used to fit the model and the validation of the results was checked by the test set. In order to classify the status of delivery (successful/unsuccessful), we compared the results from the following six techniques: LR, SVM, XGBoost, RF, NB, and LDA. Sensitivity (SE), specificity (SP), positive predictive value (PPV), negative predictive value (NPV), accuracy (ACC), area under the curve (AUC) and 95% confidence interval were used to assess the performance of the models. In order to find more reliable results, we repeated each technique 500 times. The mean ACC measures are presented.

The statistical programing R software version 3.2.3 (http://www.R-project.org) packages that included RF, NB, e1071, XGBoost, and MASS were used for data analysis. The type one error was assumed as 0.05.

### Ethical consideration

The Ethics Committee of Royan Institute (approval number: IR.ACECR.ROYAN.REC.1395.62), Tehran, Iran approved this study. The information used in this study was obtained from the data routinely registered in the patients’ medical records.

## Results

Among the assessed cycles, 4930 (81.2%) cycles resulted in successful deliveries. In the analysis, 23 variables were assessed and eight variables were summarized into four components using the principle component analysis. Finally, the association of IVF outcome and the 19 variables were evaluated. Table 1 lists the mean or frequency of the variables for both successful and unsuccessful deliveries. The unadjusted results are shown using the t test and chi-square test for continuous and categorical variables, respectively. The duration of infertility for those who delivered successfully was 0.40 years less than those with unsuccessful deliveries (t-score: 2.75, P=0.006). The mean number of previous IVF cycles was higher for cases without successful deliveries (t-score: 2.46, P=0.014). The number of injected oocytes among cases with successful deliveries was higher than those with unsuccessful deliveries (t-score:-1.99, P=0.046). Cases with successful deliveries were significantly 1.35 years younger (t-score: 8.78, P<0.001) and had 0.60 kg/m_2_ lower BMI (tscore: 4.67, P<0.001). We noted that patients with PCOS had more successful deliveries (chi-square: 6.83, degree of freedom [df]: 1, P=0.009). Male factor (chi-square: 18.25, df: 5, P=0.003), frequency of previous abortions(chi-square: 19.62, df: 2, P<0.001), and primary type of infertility (chisquare: 5.02, df: 1, P=0.025) were associated with a higher probability of successful delivery. Table 1 provides additional details of the patients’ characteristics.

**Table 1:** Patients’ characteristics in the successful and unsuccessful delivery groups


Variables	Successful deliveryn (%)				t-score or chi-square (df)	P value
	No1141 (18.8%)		Yes4930 (81.2%)			
	Mean or frequency	SD or percentage	Mean or frequency	SD or percentage		

Infertility duration (Y)	6.02	4.59	5.62	4.29	2.75	0.006
Number of previous IVF	0.94	1.27	0.85	1.15	2.46	0.014
Number of retrieved oocyte	8.36	4.16	8.56	4.19	-1.08	0.280
Number of injected oocytes	7.16	3.84	7.47	3.58	-1.99	0.046
Total number of embryos	4.77	3.00	4.94	2.87	-1.63	0.101
Number of transferred embryos	2.38	0.97	2.38	1.02	-0.19	0.848
Spermogram	3.30	3.02	3.37	3.70	-0.51	0.612
Fertilization rate	0.68	0.44	0.70	0.26	-1.28	0.199
C1	0.01	0.88	0.00	1.03	0.48	0.626
C2	0.00	0.98	0.00	1.00	0.05	0.959
C3	0.05	1.35	-0.01	0.90	1.82	0.068
C4	0.03	1.23	-0.01	0.94	1.08	0.277
Age of women (Y)					84.1(3)	<0.001
<35	882	16.90	4341	83.10		
35-37	106	20.40	414	79.60		
37-40	127	26.70	348	73.30		
>40	91	36.40	159	63.60		
Age (continuous form)	32.25	5.38	30.90	4.87	8.78	<0.001
BMI (kg/m^2^)					24.02(3)	<0.001
Underweight	14	12.30	100	87.70		
Normal	396	16.60	1990	83.40		
Overweight	446	18.60	1958	81.40		
Obese	350	22.40	1214	77.60		
BMI (continuous form)	26.53	4.26	25.93	4.14	4.67	<0.001
PCOS					6.83(1)	0.009
Yes	915	17.90	4190	82.10		
No	254	21.20	945	78.80		
Cause of infertility					18.25(5)	0.003
Female	297	19.80	1204	80.20		
Male	529	17.10	2571	82.90		
Both	155	21.30	573	78.70		
Unknown	189	19.52	779	80.48		
History of abortion					19.62(2)	<0.001
None	908	17.70	4231	82.30		
One	176	20.80	669	79.20		
≥Two	1222	25.20	362	74.80		
Infertility type					5.02(1)	0.025
Primary	813	17.80	3742	82.20		
Secondary	320	20.40	1249	79.60		
Type of cycle						0.401
ET	441	18.30	1972	81.70		
ICSI	700	19.10	2958	80.90		


C1; Number of compact and blastocysts, C2; Number of grade A and grade AB, C3; Number of early blastocysts, A compact and the day of ET, and C4; Number of AB compact, SD; Standard deviation, df; Degree of freedom, IVF; *In vitro* fertilisation, BMI; Body mass index, PCOS; Polycystic ovary syndrome, ET; Embryo transfer, and ICSI; Intracytoplasmic sperm injection.

The principle component analysis reduced eight embryo factors (number of compact, blastocyst, grade A, grade AB, early blastocyst, A compact, AB compact, and day of ET) to four components. The components were: C1 (number of compact and blastocysts); C2 (number of grade A and grade AB); C3 (number of early blastocysts, A compact and the day of ET), and C4 (number of AB compact).

The six classification methods, including NB, RF, LDA and LR, were applied. Table 2 shows a comparison of their ACC measures. Except for LR, other classification methods resulted in almost the same and high SE and PPV (SE>0.80, PPV>0.99). In contrast, the SP and NPV of LR was higher than the other approaches (SP=0.50, NPV=0.27). The total accuracy of the results showed that LR (ACC=0.64) had the worst performance where as RF (ACC=0.81) had the best performance among the six applied approaches. Moreover, the AUC for RF (AUC=60; 0.55–0.64), LDA (AUC=0.57; 0.51–0.63), LR (AUC=0.55; 0.49–0.61), and NB (AUC=0.53; 0.47–0.58) confirmed as lightly higher accuracy for RF compared to the other methods.

**Table 2:** A comparison of the six applied classification techniques using the accuracy measures


Tools	Set		Methods Tool (95% confidence interval)				
		XGBoost	SVM	NB	RF	LDA	LR

SE	Train	0.75 (0.71–0.79)	0.78 (0.75–0.81)	0.81 (0.80–0.82)	0.99 (0.98–1.00)	0.81 (0.80–0.82)	0.68 (0.67–0.69)
	Test	0.75 (0.72–0.78)	0.76 (0.73–0.79)	0.82 (0.81–0.83)	0.81 (0.80–0.82)	0.80 (0.79–0.81)	0.67 (0.66–0.68)
SP	Train	0.65 (0.62–0.68)	0.35 (0.32–0.38)	0.32 (0.31–0.33)	0.99 (0.98–1.00)	0.64 (0.61–0.67)	0.48 (0.47–0.49)
	Test	0.62 (0.56–0.70)	0.34 (0.32–0.36)	0.25 (0.22–0.28)	0.39 (0.34–0.44)	0.41 (0.35–0.47)	0.50 (0.49–0.51)
PPV	Train	0.90 (0.86–0.94)	0.60 (0.56–0.64)	0.97 (0.96–0.98)	0.99 (0.98–1.00)	0.99 (0.98–1.00)	0.85 (0.84–0.86)
	Test	0.89 (0.85–0.93)	0.58 (0.52–0.64)	0.99 (0.98–1.00)	0.99 (0.98–1.00)	0.99 (0.98–1.00)	0.84 (0.83–0.85)
NPV	Train	0.38 (0.33–0.43)	0.56 (0.53–0.59)	0.05 (0.04–0.06)	0.99 (0.98–1.00)	0.01 (0.01–0.02)	0.26 (0.25–0.27)
	Test	0.37 (0.32–0.42)	0.55 (0.53–0.57)	0.09 (0.06–0.12)	0.01 (0.01–0.02)	0.01 (0.01–0.02)	0.27 (0.26–0.28)
ACC	Train	0.74 (0.70–0.78)	0.59 (0.56–0.62)	0.79 (0.78–0.80)	0.99 (0.98–1.00)	0.81 (0.80–0.82)	0.65 (0.64–0.66)
	Test	0.73 (0.68–0.78)	0.58 (0.55–0.61)	0.77 (0.76–0.78)	0.81 (0.80–0.82)	0.80 (0.79–0.81)	0.64 (0.63–0.65)
AUC	Train	0.62 (0.57–0.67)	0.58 (0.55–0.61)	0.60 (0.56–0.64)	0.68 (0.64–0.72)	0.63 (0.58–0.68)	0.62 (0.56–0.68)
	Test	0.60 (0.57–0.63)	0.57 (0.53–0.61)	0.53 (0.47–0.58)	0.60 (0.55–0.64)	0.57 (0.51–0.63)	0.55 (0.49–0.61)


SVM; Support vector machine, XGBoost; Extreme gradient boosting, LDA; Linear discriminant analysis, LR; Logistic regression, RF; Random forest, NB; Naïve Bayes, SE; Sensitivity, SP; Specificity, PPV; Positive predictive value, NPV; Negative predictive value, ACC; Accuracy, and AUC; area under the curve.

**Fig.1:**
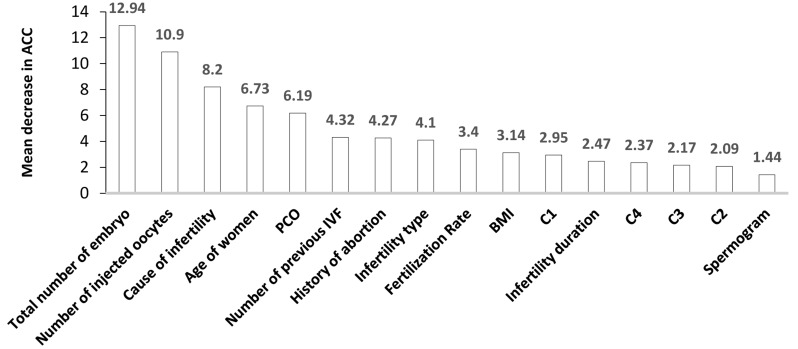
The importance of variables that affect successful delivery according to the RF approach, which had the best performance among the classification approaches. C1; Number of compact and blastocysts, C2; Number of grade A and grade AB, C3; Number of early blastocysts, A compact and the day of ET, and C4; Number of AB compact, RF; Random forest, PCOS; Polycystic ovary syndrome, IVF; *In vitro* fertilisation, BMI; Body mass index, and ACC; Accuracy.

Figure 1shows the importance of variables that affected successful delivery using the RF method. The total number of embryos, number of injected oocytes, cause of infertility, women’s age, and PCOS were the affecting predictors for a successful delivery that had a higher amount of importance in comparison to the other variables.

## Discussion

The aim of this study was to compare classical regression based methods with machine learning methods. We compared these techniques in an attempt to gain a better understanding and prediction of IVF outcomes. The application of these methods in IVF data is supposed to improve efficiency by estimating the chance of success. Generalizable and reliable prediction methods can help fulfil this purpose.

In the statistical analysis of our paper, six data mining procedures were fitted and compared to investigate successful delivery. Based on the ACC tools, the RF best fitted the data. There are several possible explanations for the better performance of the RF method in this study. First, it might be explained by the fact that modern modelling methods such as RF tend to be “data hungry” and are believed to perform better with a higher eventsper-variable ratio than classical methods (11). The larger number of continuous variables than categorical variables in this study could be another possible explanation for the better RF performance. It may also be due to the fact that tree based methods like RF account for variable interactions, while regression based methods like LR do not (12). The goodness of fit for determining a machine learning approach is a function of rates in levels of the outcome. Therefore, it does not seem to be quite rational to focus only on the total accuracy (13). A few other research indicate inconsistent performance of various classification algorithms with respect to the small/ high prevalence of the outcome (14, 15). The manner under which the predictor variables influence the result is essential for deciding the correct form of method. Therefore, discrepancies could be reported in performing classification techniques in various data areas.

Several studies have assessed machine learningbased prediction models in different outcomes during ART (e.g., embryo implantation, ongoing pregnancy, clinical pregnancy, pregnancy) (16). Uyar et al. (17) found that higher accuracy rates might be obtained by using morphological variables of individual embryos utilizing NB method for implantation prediction. Hafiz et al. (9) demonstrated that RF performed better than SVM, recursive partitioning (RPART), adaptive boosting (Adaboost), and nearest neighbour in predicting implantation outcomes of IVF and ICSI. The dataset in their study was highly unbalanced as the number of those with negative implantation was more than the positive. They explained the poor performance of SVM with the unbalanced nature of medical datasets, in particular, the one used in their study. In another study, Hassan et al. (18) compared a series of classifiers (SVM, RF, multilayer perceptron neural network [MLP], decision tree, classification and regression trees [CART] and artificial neural networks [ANN]) to predict pregnancy outcomes for IVF treatment. They reported that SVM and RF performed almost the same and both were better than the other classifiers in terms of prediction ACC and AUC. The results demonstrated that selection of a set of features for each method significantly improved the prediction ACC of pregnancy success.

The result of this study showed that the total number of embryos obtained in each cycle was associated with successful live birth. This finding supported those reported by Bartmann et al. (19), who used an artificial intelligence system to calculate pregnancy chance by taking into consideration the patients’ clinical and laboratory information. They showed that the number of embryos obtained was the best discriminant variable for pregnancy prediction; according to the artificial intelligent system developed in this study, women with more embryos tended to have greater chances for pregnancy. It was reported that the total number of embryos might be a surrogate marker for hormonal factors that act via uterine receptivity (20).

In the current study, the number of injected oocytes was another important variable that predicted IVF outcome. In a historical cohort study on 996 infertile women, modified Poisson regression analysis demonstrated that females who attained clinical pregnancy had a significantly greater number of injected oocytes compared with those who failed to achieve pregnancy (21). In another study, the number of injected oocytes was positively associated with the number of grade A embryos and could be a determinant of a successful ART (22). Zorn et al. (23) conducted a study of influencing gender characteristics of ICSI outcome in azoospermic and aspermic patients. They observed a positive association between the frequency of injected oocytes with reaching the blastocyst stage and live birth.

Cause of infertility is another important variable that affects IVF outcome, which has been confirmed by the results of numerous similar studies (24). Nelson and Lawlor (25) predicted live birth and weight at birth among infants born from IVF. They observed that male cause of infertility was linked to lower chances of successful pregnancy in patients who did not receive ICSI. Factors associated with failed treatment were evaluated by Bhattacharya et al. (26); the results showed that the risk of poor fertilization was more common among patients with tubal disease, male factor, and endometriosis. Moreover, they noted that the risk of non-live births among those with tubal disease and male factor was higher than those with unexplained infertility. It has been demonstrated that cause of infertility plays a role in determining poor intermediate outcomes. Elizur et al. (27) investigated the predictive factors for IVF treatment pregnancy results; they observed that delivery rate among those with male factor was significantly higher than other aetiologies. 

A woman’s age was another significant factor for achieving a successful pregnancy. It has been widely debated that with increasing of female age, the IVF outcomes become increasingly worse. Among infertile cases, the Society for ART (SART) stated that 47% of ETs among women younger than 35 years of age resulted in successful delivery. The proportion was 38% for ages 35–37, 28% for ages 38–40, 16% for ages 41–42 and 6% for older than 42 years of age (28). Nazemian et al. (29) investigated the impact of age on IVF outcome. They reported that cases younger than 25 years of age have lower fertilization rates as well as a decreased frequency of high quality embryos. In their study, clinical pregnancy and implantation rates were similar to those who were 30-35years of age. In another research, Yan et al. (30) evaluated the mechanism by which maternal age affects the outcomes of IVF cases. Patients older than 40 years had a disadvantaged IVF outcome and increased numbers of miscarriages.

The current work shows that PCOS is a potential influencing factor for live birth. Beydoun et al. (31) have reported that PCOS has a distinct effect on the early stages of pregnancy among women who undergo IVF/ICSI, but not on the later stages. Ryan et al. (32), in a study of a large number of infertile women, showed that women with PCOS had increased odds for childbirth. Moreover, PCOS significantly confounded the relationship between the duration of ovarian stimulation and treatment success. Earlier findings also showed a greater number of oocytes were retrieved in PCOS women compared to women without PCOS (31), and greater number of follicles>16 mm and MII oocytes in PCOS women compared to women with subfertile male partners and those with unexplained infertility (33). These results imply a higher amount of ovarian capacity in PCOS women and the compensatory impact of this capacity (34, 35). However, the results from a large number of studies mentioned that the role of PCOS in ART success mainly depended on obesity, insulin resistance, and other metabolic syndrome features (36, 37).

This study had several limitations. First, this research was carried out in one infertility clinic and this limits the generaliz ability of our findings. Second, other predictors such as basal FSH and somegenetic features are potential factors that were not recorded by the Centre (38, 39). Third, the distribution of IVF outcome was not balanced (unbalanced dataset). Fourth, the AUCs were relatively small and the performance of the models was compared using accuracy tools in conjunction with the AUC.

RF performance has less dependence on parameter values than other machine learning methods. However, in future investigations it might be possible to achieve more improvements in this method by using optimization procedures to simultaneously tune the RF parameters or use RF based on conditional inference trees to address the problem of variable selection (40).

Results obtained from machine learning could help to determine the risk factors and their impact in real world settings. It could also help to predict the personalized chance of an ART outcome before the treatment procedure. This would assist clinicians decide whether it is worth to startan ART procedure and would also provide infertile couples information about the chances for success.

## Conclusion

This study sought to classify IVF successful delivery based on six machine learning approaches: SVM, XGBoost, LDA, LR, RF and NB by using couples’ characteristics and available data on oocytes, sperm, and embryos. This study indicated that successful delivery after ART is strongly dependent on various characteristics of the patients, which included total number of embryos, number of injected oocytes, cause of infertility, age of women, and PCOS. Our results indicated that the RF approach could be a better choice to classify ART outcome among other classification methods. These results could assist clinicians to have a better prediction and management of ART treatment and advise patients accordingly.
